# Acute Histological Chorioamnionitis and Birth Weight in Pregnancies With Preterm Prelabor Rupture of Membranes: A Retrospective Cohort Study

**DOI:** 10.3389/fphar.2022.861785

**Published:** 2022-03-04

**Authors:** Jana Matulova, Marian Kacerovsky, Helena Hornychova, Jaroslav Stranik, Jan Mls, Richard Spacek, Hana Burckova, Bo Jacobsson, Ivana Musilova

**Affiliations:** ^1^ Department of Non-Medical Studies, Charles University, Faculty of Medicine in Hradec Kralove, Hradec Kralove, Czechia; ^2^ Department of Obstetrics and Gynecology, University Hospital Hradec Kralove, Charles University, Faculty of Medicine in Hradec Kralove, Hradec Kralove, Czechia; ^3^ Biomedical Research Center, University Hospital Hradec Kralove, Hradec Kralove, Czechia; ^4^ Fingerland’s Institute of Pathology, University Hospital Hradec Kralove, Charles University, Hradec Kralove, Czechia; ^5^ Department of Obstetrics and Gynecology, University Hospital Ostrava, Ostrava, Czechia; ^6^ Department of Neonatology, University Hospital Ostrava, Ostrava, Czechia; ^7^ Department of Obstetrics and Gynecology, Institute of Clinical Science, Sahlgrenska Academy, University of Gothenburg, Gothenburg, Sweden; ^8^ Region Västra Götaland, Sahlgrenska University Hospital, Department of Obstetrics and Gynecology, Gothenburg, Sweden; ^9^ Department of Genetics and Bioinformatics, Domain of Health Data and Digitalization, Institute of Public Health, Oslo, Norway

**Keywords:** amnion, intergrowth, neutrophils, placenta, preterm delivery

## Abstract

**Aim:** To assess the association between the birth weight of newborns from pregnancies with preterm prelabor rupture of membranes (PPROM) and the presence of acute histological chorioamnionitis (HCA) with respect to the: i) fetal and maternal inflammatory responses and ii) acute inflammation of the amnion.

**Material and Methods:** This retrospective cohort study included 818 women with PPROM. A histopathological examination of the placenta was performed. Fetal inflammatory response was defined as the presence of any neutrophils in umbilical cord (histological grades 1–4) and/or chorionic vasculitis (histological grade 4 for the chorionic plate). Maternal inflammatory response was defined as the presence of histological grade 3–4 for the chorion-decidua and/or grade 3 for the chorionic plate and/or grade 1–4 for the amnion. Acute inflammation of the amnion was defined as the presence of any neutrophils in the amnion (histological grade 1–4 for the amnion). Birth weights of newborns were expressed as percentiles derived from INTERGROWTH-21st standards for the i) estimated fetal weight and ii) newborn birth weight.

**Results:** No difference in percentiles of birth weights of newborns was found among the women with the women with HCA with fetal inflammatory response, with HCA with maternal inflammatory response and those without HCA. Women with HCA with acute inflammation of the amnion had lower percentiles of birth weights of newborns, derived from the estimated fetal weight standards, than women with HCA without acute inflammation of the amnion and those with the absence of HCA in the crude (with acute inflammation: median 46, without acute inflammation: median 52, the absence of HCA: median 55; *p* = 0.004) and adjusted (*p* = 0.02) analyses. The same subset of pregnancies exhibited the highest rate of newborns with a birth weight of ≤25 percentile. When percentiles were derived from the newborn weight standards, no differences in birth weights were observed among the subgroups.

**Conclusion:** Acute inflammation of the amnion was associated with a lower birth weight in PPROM pregnancies, expressed as percentiles derived from the estimated fetal weight standards.

## Introduction

Preterm prelabor rupture of the membranes (PPROM) is defined as the rupture of fetal membranes with leakage of amniotic fluid before the onset of regular uterine activity prior to 37 weeks of gestational age (Mercer, 2003; Mercer, 2005). PPROM represents a phenotype of spontaneous preterm delivery that complicates approximately 3–4% of all pregnancies (Mercer, 2003; Mercer, 2005). Despite its predominantly non-infectious nature, PPROM might be complicated by the presence of acute inflammatory changes in the amniotic fluid and/or acute inflammatory lesions of the placenta (Cobo et al., 2012; Musilova et al., 2015).

The presence of acute inflammatory lesions of the placenta is characterized by diffuse infiltration of neutrophils in any of the structures of the placenta (fetal membranes, the placental disc, and the umbilical cord) and collectively are called acute histological chorioamnionitis (HCA) (Kim et al., 2015). Depending on the primary source of infiltrating neutrophils, HCA can be divided into the following: i) maternal inflammatory response, when extravasating maternal neutrophils infiltrate fetal membranes and/or the chorionic plate and ii) fetal inflammatory response, when extravasating fetal neutrophils invade the vessels of the chorionic plate and/or in structures of the umbilical cord (Kim et al., 2015).

The fetal inflammatory response is known to be the most severe form of HCA that is associated with adverse neonatal outcomes (Kim et al., 2015). It is also considered a histopathological counterpart of the fetal inflammatory response syndrome (Pacora et al., 2002). Apart from the fetal inflammatory response, a specific subtype of HCA, infiltration of the amnion by neutrophils has been shown to be related to very intense inflammatory responses, measured by various markers in the amniotic fluid and umbilical cord blood, regardless of the concurrent presence or absence of funisitis (Park et al., 2009). Therefore, two subtypes of HCA–with a fetal inflammatory response and acute inflammation of the amnion, should be of utmost clinical interest since they are associated with the most severe inflammatory responses.

It is obvious that placental lesions other than HCA (maternal and fetal vascular malperfusion, placental hemorrhage, and chronic villitis) mainly lead to impaired placental functions, which can be followed by an alteration of fetal growth (Salafia et al., 1989; Salafia et al., 1992; Salafia et al., 1995; Tyson and Staat, 2008; Mifsud and Sebire, 2014; Novac et al., 2018; Aviram et al., 2019). Collectively, these lesions represent underlying pathologies for conditions known as either small-for-gestational-age (SGA) or fetal growth restriction (FGR).

Nevertheless, some studies have provided evidence for the relationship between impaired fetal growth and HCA (Williams et al., 2000; Levy et al., 2021). This unexpected association is further supported by the following observations: i) lower birth weight is associated with upregulation of genes encoding proinflammatory transcription factor activator protein-1 (Ross et al., 2019); ii) the presence of HCA is found in approximately 10% of SGA pregnancies (DiGiulio et al., 2010); iii) a higher number of placental macrophages and increased placental inflammatory profile are found in pregnancies with impaired fetal growth or FGR (Street et al., 2006; Street et al., 2008; Sharps et al., 2020); iv) elevated concentrations of inflammatory markers in umbilical cord blood may be found in SGA newborns (Amarilyo et al., 2011; Lausten-Thomsen et al., 2014).

However, there is a shortage of information on whether the presence of HCA, particularly its most severe forms, with fetal inflammatory response and acute inflammation of the amnion, is related to impaired fetal growth in pregnancies complicated by PPROM. To fill this knowledge gap, a study on women with singleton pregnancies complicated by PPROM was conducted with the following goals: i) to assess birth weight, expressed as percentiles, and to compare the rates of the percentiles of birth weight that are less than or equal to the first, 10th, and 25th percentiles with respect to the presence of HCA with fetal and maternal inflammatory responses and the absence of HCA; ii) to assess birth weight, expressed as percentiles, and to compare the rates of the percentiles of birth weight that are less than or equal to the first, 10th, and 25th percentiles with respect to the presence of HCA with and without acute inflammation of the amnion and the absence of HCA; and iii) to assess birth weight, expressed as percentiles, and to compare the rates of the percentiles of birth weight that are less than or equal to the first, 10th, and 25th percentiles with respect to the severity of acute inflammation of the amnion.

## Methods

This study was a retrospective cohort study conducted in pregnant women with PPROM admitted to the Department of Obstetrics and Gynecology, University Hospital Hradec Kralove in the Czech Republic between May 2008 and March 2021 who met the following criteria: i) singleton pregnancy; ii) gestational age at admission between 24 + 0 weeks and 36 + 6 weeks; iii) maternal age ≥18 years; iv) available histopathological results of the placenta. The exclusion criteria were as follows: i) pregnancy-related complications such as gestational diabetes, gestational hypertension, or preeclampsia; ii) chronic diseases such as pregestational diabetes and chronic hypertension; iii) structural or chromosomal abnormalities of the fetus.

Gestational age was determined based on the first-trimester ultrasound scan. The diagnosis of PPROM was established based on visual confirmation of amniotic fluid pooling in the posterior vaginal fornix by a sterile speculum examination. If uncertainty persisted after the clinical examination, the leakage of amniotic fluid was confirmed or ruled out using a test to determine the presence of insulin-like growth factor-binding protein in the vaginal fluid (Actim PROM test; Medix Biochemica, Kauniainen, Finland).

Women with PPROM at less than 34 weeks of gestation were treated with antibiotics and corticosteroids to accelerate lung maturation. Tocolytics were used only when regular uterine activity appeared during the course of corticosteroids, but not for longer than 48 h. Women with PPROM beyond 34 weeks of gestation were treated with antibiotics only. The women included in this study were managed using two different approaches. Between May 2008 and December 2013, women were treated actively (except those at < 28 gestational weeks). Labor was induced, or an elective cesarean section was performed after finalizing corticosteroid treatment but no later than 72 h after the rupture of the membranes, depending on the gestational age, fetal status, and maternal serum C-reactive protein concentrations. Since January 2014, the performance of transabdominal amniocentesis to assess the status of the intra-amniotic environment (microbial invasion of the amniotic cavity and intra-amniotic inflammation) has been a routine part of the clinical management of women with PPROM (Musilova et al., 2017). Thus, women admitted between January 2014 and May 2021 were managed differently. Women with intra-amniotic infection (the presence of both microbial invasion of the amniotic cavity and intra-amniotic inflammation) beyond the 28th gestational week were managed actively (labor was induced, or an elective cesarean section was performed after finalizing corticosteroid treatment within 72 h of membrane rupture for pregnancies before 34 weeks of gestation and within 24 h of membrane rupture for those beyond 34 weeks). The remaining women with PPROM were managed expectantly (Musilova et al., 2017).

After delivery, the placenta, fetal membranes, and umbilical cord were fixed in 10% neutral buffered formalin. Tissue samples were obtained from the placenta (at least two samples), fetal membranes (one sample from the free margin of membranes, one from the central part of the membranes, and one from the membranes with a marginal part of the placenta), and umbilical cord (usually one sample), which were routinely processed and embedded in paraffin. Sections of the tissue blocks were stained with hematoxylin and eosin.

The degree of neutrophil infiltration was evaluated separately in the free membranes (amnion and chorion-decidua), chorionic plate, and umbilical cord based on the criteria provided by Salafia et al. (Salafia et al., 1989). Histopathological examinations were performed by a single pathologist (HH) who was blinded to the clinical status of the women.

The collection of clinical samples and information was approved by the Ethics Committee of the University Hospital of Hradec Kralove, Czech Republic (19 March 2008; No. 200804 SO1P, which was renewed in July 2014 and January 2019, decisions No. 201407 S14P and No. 201902 S16P, respectively). Written informed consent was obtained from all the participants. Biological samples (amniotic fluid, cervical fluid, and umbilical cord blood) from the women included in this study were used in our previous studies and are presented in the publications. A total of 528 women from this cohort were included in our previous publication, where an association between birth weight and microbial invasion of the amniotic cavity and/or intra-amniotic inflammation was evaluated (Matulova et al., 2021). All methods used in this study were carried out in accordance with the relevant guidelines and regulations.

### Birth Weight Percentiles

All newborns were weighed immediately after birth using a calibrated electronic scale. Birth weights were converted to percentiles derived from the INTERGROWTH-21st standards (Villar et al., 2014a; Villar et al., 2014b; Papageorghiou et al., 2014; Stirnemann et al., 2017) for the: i) estimated fetal weight (Papageorghiou et al., 2014; Stirnemann et al., 2017) and ii) newborn birth weight (Villar et al., 2014a).

### Clinical Definitions

HCA was diagnosed based on the histological grade 3-4 for the chorion-decidua and/or grade 3-4 for the chorionic plate and/or grade 1–4 for the umbilical cord and/or grade 1–4 for the amnion (Salafia et al., 1989). Based on the type of the inflammatory response, women with the presence of HCA were further subdivided into those with: i) fetal inflammatory response—the presence of histological grade 1–4 for the umbilical cord (any neutrophils present in the umbilical cord) and/or histological grade 4 for the chorionic plate (chorionic vasculitis) and ii) maternal inflammatory response—the presence of histological grade 3-4 for the chorion-decidua and/or grade 3 for the chorionic plate and/or grade 1–4 for the amnion. Based on the presence or absence of acute inflammation of the amnion, women with the presence of HCA were further divided into those: i) with acute inflammation of the amnion–the presence of histological grade 1-4 for the amnion and ii) without acute inflammation of the amnion–the presence of histological grade 3-4 for the chorion-decidua and/or grade 3-4 for the chorionic plate and/or grade 1–4 for the umbilical cord (Salafia et al., 1989). Severity of acute inflammation of the amnion: grade 1—one focus of at least five neutrophils; grade 2—more than grade 1 focus or at least one focus of 5–20 neutrophils; grade 3—multiple and/or confluent grade 2 foci; and grade 4—diffuse and dense acute inflammation (Salafia et al., 1989).

### Statistical Analysis

The normality of the data was tested using the Anderson-Darling test. Continuous variables were compared using the nonparametric Jonckheere-Terpstra test for trend, or Mann-Whitney *U* test, as appropriate, and presented as medians [interquartile range (IQR)]. Categorical variables were compared using the Cochran-Armitage test for trend, and presented as numbers (%). Spearman’s partial correlation was used to adjust the results for the following potential confounders: various methods for managing PPROM, maternal age, nulliparity, smoking, the interval between PPROM and amniocentesis, the interval between amniocentesis and delivery, administration of corticosteroids, mode of delivery. Differences were considered significant at *p* < 0.05. All *p*-values were obtained using two-tailed tests. All statistical analyses were performed using GraphPad Prism version 8.4.3 and the Statistical Package for the Social Sciences (SPSS), version 28.0.0.0, for Windows (SPSS Inc., Chicago, IL, United States).

## Results

A total of 918 women with singleton pregnancies complicated by PPROM were eligible for the study, and 100 women were excluded for the following reasons: i) gestational diabetes mellitus (*n* = 53); ii) gestational hypertension (*n* = 19); iii) preeclampsia (*n* = 5); iv) pre-gestational diabetes mellitus (*n* = 12); v) chronic hypertension (*n* = 5); vi) combination of the above-mentioned diseases (*n* = 6). The remaining 818 women were included in the analysis.

In total, HCA was observed in 494 (60%) women. Among women with the presence of HCA, fetal and maternal inflammatory responses were identified in 343 (69%) and 151 (31%) women, respectively. Remaining 324 (60%) women had the absence of HCA. The demographic and clinical characteristics of the study population, as well as short-term neonatal outcomes, with respect to the presence of fetal and maternal inflammatory responses and the absence of HCA are shown in Table 1. Among women with the presence of HCA, 279 (57%) and 215 (43%) women were with and without acute inflammation of the amnion was observed in 279 (34%) women. The demographic and clinical characteristics of the study population, as well as short-term neonatal outcomes, with respect to the presence of HCA with and without inflammation of the amnion and the absence of HCA are shown in Table 2.

**TABLE 1:** Maternal and clinical characteristics of women with preterm prelabor rupture of membranes and short-term neonatal outcomes with respect to the presence of HCA with fetal and maternal inflammatory responses and the absence of HCA.

Characteristic	The presence of HCA		The absence of HCA (*n* = 324)	*p-*value
	With fetal inflammatory response (*n* = 343)	With maternal inflammatory response (*n* = 151)		
Maternal age [years, median (IQR)]	31 (27–36)	31 (27–35)	30 (27–34)	**0.05**
Primiparous [number (%)]	153 (45%)	81 (54%)	196 (61%)	**<0.0001**
Pre-pregnancy body mass index [kg/m^2^, median (IQR)]	23.0 (20.6–26.7)	23.7 (21.1–26.7)	22.4 (20.3–26.0)	0.23
Gestational age at sampling [weeks + days, median (IQR)]	32 + 4 (30 + 0–34 + 4)	33 + 3 (31 + 2–35 + 0)	34 + 4 (32 + 3–35 + 4)	**<0.0001**
Gestational age at delivery [weeks + days, median (IQR)]	33 + 1 (30 + 5–35 + 0)	33 + 6 (32 + 0–35 + 3)	34 + 5 (33 + 0–35 + 5)	**<0.0001**
Latency from PPROM to AMC [hours, median (IQR)]	5 (3–11)	5 (3–9)	4 (3–8)	**0.02**
Latency from AMC to delivery [hours, median (IQR)]	64 (25–128)	53 (22–101)	26 (13–60)	**<0.0001**
Active management of PPROM [number (%)]	106 (31%)	55 (36%)	131 (40%)	**0.01**
CRP levels at admission [mg/L, median (IQR)]	6.2 (3.1–11.3)	5.8 (3.2–8.6)	5.0 (2.4–9.1)	**0.006**
WBC count at admission [x10^9^ L, median (IQR)]	12.3 (10.4–15.3)	12.0 (10.4–14.8)	12.1 (10.0–14.6)	0.10
Smoking [number (%)]	69 (20%)	24 (16%)	45 (14%)	**0.03**
Administration of corticosteroids [number (%)]	272 (79%)	106 (70%)	189 (58%)	**<0.0001**
Administration of antibiotics [number (%)]	338 (99%)	150 (99%)	316 (98%)	0.32
Spontaneous vaginal delivery [number (%)]	217 (63%)	104 (69%)	240 (74%)	**0.003**
Cesarean section [number (%)]	125 (36%)	45 (30%)	80 (25%)	**0.001**
Forceps delivery [number (%)]	1 (1%)	2 (1%)	4 (1%)	0.19
Sex of the newborn (female) [number (%)]	171 (50%)	62 (41%)	145 (45%)	0.18
Birth weight [grams, median (IQR)]	1960 (1460–2390)	2090 (1680–2520)	2290 (1930–2610)	**<0.0001**
Apgar score <7; 5 min [number (%)]	15 (4%)	6 (4%)	4 (1%)	**0.02**
Apgar score <7; 10 min [number (%)]	5 (2%)	3 (2%)	1 (1%)	0.16
Transient tachypnea of newborns [number (%)]	13 (4%)	1 (1%)	8 (3%)	0.28
Respiratory distress syndrome [number (%)]	117 (34%)	44 (29%)	70 (22%)	**0.0003**
Bronchopulmonary dysplasia [number (%)]	38 (11%)	10 (7%)	5 (2%)	**<0.0001**
Need for intubation [number (%)]	37 (11%)	11 (7%)	10 (3%)	**0.0001**
Intraventricular hemorrhage (grades I-II) [number (%)]	54 (16%)	18 (12%)	51 (16%)	0.99
Intraventricular hemorrhage (grades III-IV) [number (%)]	7 (2%)	2 (1%)	0 (0%)	**0.02**
Retinopathy of prematurity [number (%)]	20 (6%)	5 (3%)	3 (1%)	**0.0005**
Necrotizing enterocolitis [number (%)]	10 (3%)	2 (1%)	1 (0%)	**0.007**
Early-onset sepsis [number (%)]	30 (9%)	3 (2%)	4 (1%)	**<0.0001**
Late-onset sepsis [number (%)]	13 (4%)	3 (2%)	5 (2%)	0.07
Compound neonatal morbidity [number (%)]	165 (48%)	55 (36%)	100 (31%)	**<0.0001**
Neonatal death [number (%)]	6 (2%)	2 (1%)	2 (1%)	0.18

*p-*value: comparison among the women with the presence of HCA with fetal inflammatory response, women with the presence of HCA with maternal inflammatory response, and women with the absence of HCA. Continuous variables were compared using a nonparametric Jonckheere-Terpstra test for trend and presented as median (interquartile range). Categorical variables were compared using Cochran-Armitage test for trend a presented as number (%). Statistically significant results are marked in bold.

AMC, amniocentesis; CRP, C-reactive protein; HCA, acute histological chorioamnionitis; IQR, interquartile range; PPROM, preterm prelabor rupture of membranes; WBC, white blood cells.

**TABLE 2:** Maternal and clinical characteristics of women with preterm prelabor rupture of membranes and short-term neonatal outcomes with respect to the presence of HCA with and without acute inflammation in the amnion and the absence of HCA.

Characteristic	The presence of HCA		The absence of HCA (n = 324)	*p-*value
	With acute inflammation of the amnion (n = 279)	Without acute inflammation of the amnion (n = 215)		
Maternal age [years, median (IQR)]	31 (27–35)	32 (28–35)	30 (27–34)	0.07
Primiparous [number (%)]	117 (42%)	117 (54%)	196 (61%)	**<0.0001**
Pre-pregnancy body mass index [kg/m^2^, median (IQR)]	23.0 (20.6–27.1)	23.5 (21.0–26.3)	22.4 (20.3–26.0)	0.22
Gestational age at sampling [weeks + days, median (IQR)]	31 + 5 (28+6-34 + 0)	34 + 0 (32+0-35 + 2)	34 + 4 (32 + 3 – 35 + 4)	**<0.0001**
Gestational age at delivery [weeks + days, median (IQR)]	32 + 2 (29+4-34 + 2)	34 + 2 (32+4-35 + 4)	34 + 5 (33 + 0 – 35 + 5)	**<0.0001**
Latency from PPROM to AMC [hours, median (IQR)]	6 (3–12)	5 (3–8)	4 (3–8)	**0.001**
Latency from AMC to delivery [hours, median (IQR)]	65 (29–142)	46 (19–96)	26 (13–60)	**<0.0001**
Active management of PPROM [number (%)]	97 (35%)	64 (30%)	131 (40%)	0.13
CRP levels at admission [mg/L, median (IQR)]	6.1 (2.9–11.4)	5.9 (3.3–9.0)	5.0 (2.4–9.1)	**0.03**
WBC count at admission [x10^9^ L, median (IQR)]	12.5 (10.7–15.3)	12.1 (9.9–14.6)	12.1 (10.0–14.6)	0.06
Smoking [number (%)]	55 (20%)	38 (18%)	45 (14%)	0.06
Administration of corticosteroids [number (%)]	229 (82%)	149 (69%)	189 (58%)	**<0.0001**
Administration of antibiotics [number (%)]	275 (99%)	213 (99%)	316 (98%)	0.31
Spontaneous vaginal delivery [number (%)]	171 (61%)	150 (70%)	240 (74%)	**0.0008**
Cesarean section [number (%)]	107 (38%)	63 (29%)	80 (25%)	**0.0003**
Forceps delivery [number (%)]	1 (1%)	2 (1%)	4 (1%)	0.25
Sex of the newborn (female) [number (%)]	133 (48%)	100 (47%)	145 (45%)	0.47
Birth weight [grams, median (IQR)]	1850 (1300–2260)	2220 (1870–2580)	2290 (1930–2610)	**<0.0001**
Apgar score <7; 5 min [number (%)]	18 (6%)	3 (1%)	4 (1%)	**0.0003**
Apgar score <7; 10 min [number (%)]	6 (2%)	2 (1%)	1 (1%)	**0.03**
Transient tachypnea of newborns [number (%)]	7 (3%)	7 (3%)	8 (3%)	0.96
Respiratory distress syndrome [number (%)]	108 (39%)	53 (25%)	70 (22%)	**<0.0001**
Bronchopulmonary dysplasia [number (%)]	39 (14%)	9 (4%)	5 (2%)	**<0.0001**
Need for intubation [number (%)]	39 (14%)	9 (4%)	10 (3%)	**<0.0001**
Intraventricular hemorrhage (grades III-IV) [number (%)]	47 (17%)	25 (12%)	51 (16%)	0.75
Intraventricular hemorrhage (grades III-IV) [number (%)]	7 (3%)	2 (1%)	0 (0%)	**0.003**
Retinopathy of prematurity [number (%)]	21 (8%)	4 (2%)	3 (1%)	**<0.0001**
Necrotizing enterocolitis [number (%)]	11 (4%)	1 (1%)	1 (0%)	**0.0005**
Early-onset sepsis [number (%)]	24 (9%)	9 (4%)	4 (1%)	**<0.0001**
Late-onset sepsis [number (%)]	13 (5%)	3 (1%)	5 (2%)	**0.02**
Compound neonatal morbidity [number (%)]	145 (52%)	75 (35%)	100 (31%)	**<0.0001**
Neonatal death [number (%)]	6 (2%)	2 (1%)	2 (1%)	0.09

*p-*value: comparison among the women with the presence of HCA with acute inflammation in the amnion, women with the presence of HCA without acute inflammation in the amnion, and women with the absence of HCA. Continuous variables were compared using a nonparametric Jonckheere-Terpstra test for trend and presented as median (interquartile range). Categorical variables were compared using Cochran-Armitage test for trend a presented as number (%). Statistically significant results are marked in bold.

AMC, amniocentesis; CRP, C-reactive protein; HCA, acute histological chorioamnionitis; IQR, interquartile range; PPROM, preterm prelabor rupture of membranes; WBC, white blood cells.

### Birth Weight Expressed as Percentiles Derived From the Estimated Fetal Weight Standards

**FIGURE 1:**
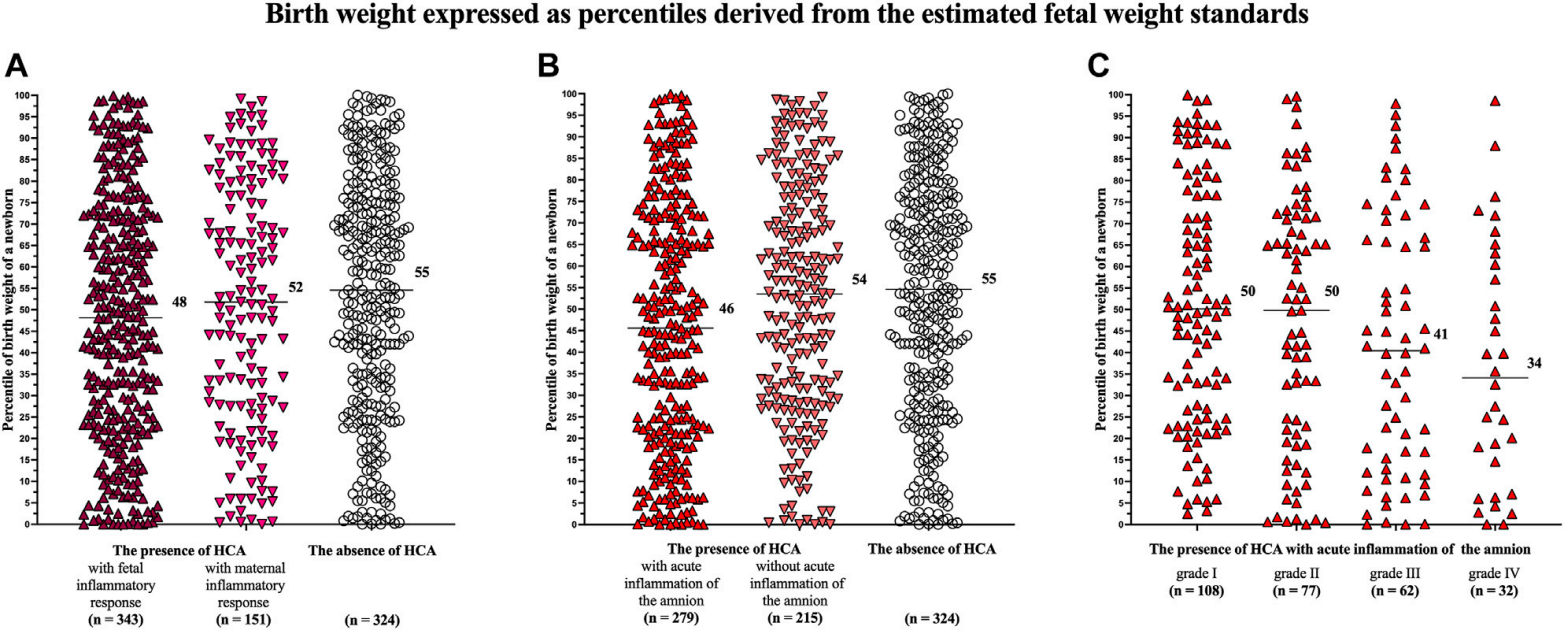
Comparison of the birth weights of newborns, expressed as percentiles derived from the estimated fetal weight standards, among the women with the presence of HCA with fetal and maternal inflammatory responses and those with the absence of HCA **(A)**; among the women with the presence of HCA with and without acute inflammation of the amnion and those with the absence of HCA **(B)**; and among the women with of acute inflammation of the amnion divided based on its severity (grades I–IV) **(C)**. A difference was observed among the women with the presence of HCA with fetal and maternal inflammatory responses and those with the absence of HCA only in the crude analysis (*p =* 0.02) but not in the adjusted analysis (*p* = 0.14) **(A)**. A difference was observed among the women with the presence of HCA with and without acute inflammation and those with the absence of HCA in both the crude and adjusted analyses (*p* = 0.004, *p* = 0.02) **(B)**. A difference was observed among the women with acute inflammation of the amnion divided into the subgroups based on its severity in both the crude and adjusted analyses (*p* = 0.004, *p* = 0.03) **(C)**. Medians are marked. Abbreviation: HCA, acute histological chorioamnionitis.

A difference in the percentiles of birth weights of newborns was identified among the women with the presence of HCA with fetal (median 48, IQR 24–72) and maternal (median 52, IQR 28–78) inflammatory responses and those with the absence of HCA (median 55, IQR 31–76) in the crude analysis (*p* = 0.02; Figure 1A) but not in the analysis adjusted for potential confounders (*p* = 0.14).

Women with the presence of HCA with acute inflammation of the amnion had lower percentiles of birth weights of newborns (median 46, IQR 21–71) than women with the presence of HCA without acute inflammation of the amnion (median 54, IQR 30–76) and those with the absence of HCA (median 55, IQR 31–76) in the crude (*p* = 0.004; Figure 1B) and adjusted (*p* = 0.02) analyses (Table 3).

**TABLE 3:** Comparisons of the percentiles of birth weights of newborns derived from the estimated fetal weight standards among the subgroups of women with the presence of HCA with and without acute inflammation of the amnion and those with the absence of HCA.

	HCA with acute inflammation of the amnion	HCA without acute inflammation of the amnion	The absence of HCA
**HCA with acute inflammation of the amnion**	X	***p* = 0.01 adj. *p* = 0.03**	***p =* 0.002 adj. *p* = 0.03**
**HCA without acute inflammation of the amnion**	***p* = 0.01 adj. *p* = 0.03**	x	*p* = 0.31
**The absence of HCA**	***p =* 0.002 adj. *p* = 0.03**	*p* = 0.31	x

*p*-value: a comparison between two subgroups (a nonparametric Mann-Whitney *U* test). adj. *p*-value: a comparison between two subgroups after the adjustment for gestational potential confounders (a Spearman partial correlation). Statistically significant results are marked in bold.

HCA, acute histological chorioamnionitis.

A difference in the percentiles of birth weights of newborns was found among the women with the presence of HCA with acute inflammation of the amnion, when the women were divided into four subgroups based on the severity of acute inflammation of the amnion [grade I (median 50, IQR 25–77), grade II (median 50, IQR 20–72), grade III (median 41, IQR 13–68), and grade IV (median 37, IQR 9–63)] in the crude (*p* = 0.004; Figure 1C) and adjusted (*p* = 0.03) analyses.

A difference in the rates of the birth weights of newborns that were less than or equal to the 25th percentiles were observed among women with the presence of HCA with fetal and maternal inflammatory responses and those with the absence of HCA (*p* = 0.05; Table 4), as well as and among women with the presence of HCA with and without acute inflammation of the amnion and those with the absence of HCA (*p* = 0.0009; Table 4). However, after adjustment for potential confounders, only the latter result remained significant (*p* = 0.29; *p* = 0.02). Differences in the rates of the birth weights of newborns that were less than or equal to the first, 10th, and 25th percentiles were observed among women with HCA with acute inflammation of the amnion, when the women were stratified into the four subgroups based on the severity of acute inflammation of the amnion in the crude (first percentile: *p* = 0.02; 10th percentile: *p* = 0.003; 25th percentile: *p* = 0.03; Table 4) and adjusted analyses (first percentile: *p* = 0.02; 10th percentile: *p* = 0.01; 25th percentile: *p* = 0.03; Table 4).

**TABLE 4:** The rate of newborns with birth weights, expressed as percentiles derived from the estimated fetal weight standards, that were less than or equal to the first, 10th, and 25th percentiles according to: (a) the presence of HCA with fetal and maternal inflammatory responses and the absence of HCA; (b) the presence of HCA with and without acute inflammation of the amnion and the absence of HCA; and (c) severity of acute inflammation of the amnion (grades I-IV).

	≤1 percentile		≤10 percentiles		≤25 percentiles	
a)						
The presence of HCA with fetal inflammatory response (*n* = 343)	12 (4%)	*p* = 0.44	37 (11%)	*p* = 0.51	91 (27%)	***p* = 0.05**
The presence of HCA with maternal inflammatory response (*n* = 151)	5 (3%)		16 (11%)		34 (23%)	
The absence of HCA (*n* = 324)	8 (3%)		30 (10%)		65 (20%)	
b)						
The presence of HCA with acute inflammation of the amnion (*n* = 279)	10 (4%)	*p* = 0.57	38 (14%)	*p* = 0.82	89 (32%)	***p* = 0.0009** ^a^
The presence of HCA without acute inflammation of the amnion (*n* = 215)	7 (3%)		15 (7%)		36 (17%)	
The absence of HCA (*n* = 324)	8 (3%)		30 (10%)		65 (20%)	
c)						
Acute inflammation of the amnion—grade I (*n* = 108)	0 (0%)	***p* = 0.02** ^a^	7 (7%)	***p* = 0.003** ^a^	28 (26%)	***p* = 0.028** ^a^
Acute inflammation of the amnion—grade II (*n* = 77)	4 (5%)		12 (16%)		24 (31%)	
Acute inflammation of the amnion—grade III (*n* = 62)	4 (7%)		11 (18%)		24 (38%)	
Acute inflammation of the amnion—grade IV (*n* = 32)	2 (6%)		8 (25%)		14 (44%)	

Variables are presented as number (%) and were compared using Cochran-Armitage test for trend. Statistically significant results are marked in bold.

HCA, acute histological chorioamnionitis.

a Result remains significant after the adjustment for potential confounders.

### Birth Weight Expressed as Percentiles Derived From the Newborn Birth Weight Standards

**FIGURE 2:**
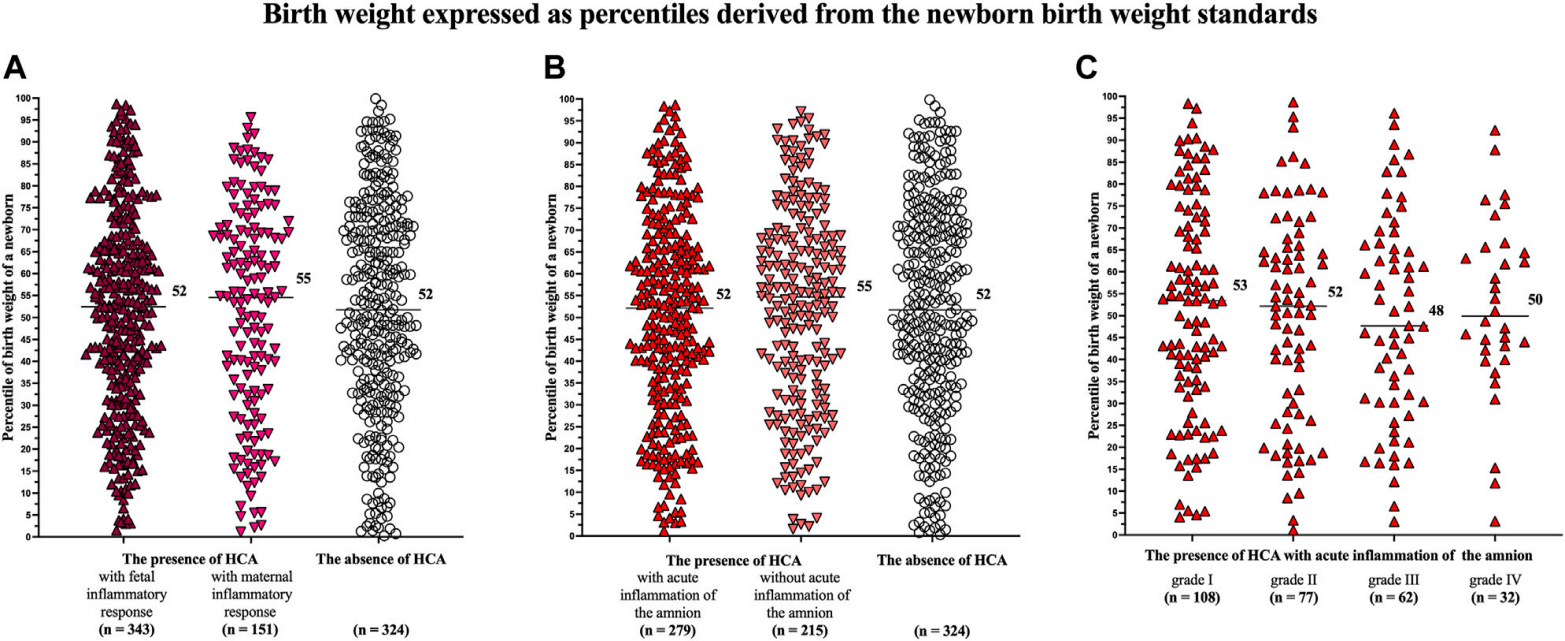
Comparison of the birth weights of newborns, expressed as percentiles derived from the newborn birth weight standards, among the women with the presence of HCA with fetal and maternal inflammatory responses and those with the absence of HCA **(A)**; among the women with the presence of HCA with and without acute inflammation of the amnion and those with the absence of HCA **(B)**; and among the women with acute inflammation of the amnion divided based on its severity (grades I-IV) **(C)**. No difference was observed among the women with the presence of HCA with fetal and maternal inflammatory responses and those with the absence of HCA in the crude analysis (*p* = 0.56) **(A)**. No difference was observed among the women with the presence of HCA with and without acute inflammation of the amnion and those with the absence of HCA in the crude analysis (*p* = 0.41) **(B)**. No difference was observed among the women with acute inflammation of the amnion divided into the subgroups based on the severity of acute inflammation of the amnion (*p* = 0.85). Medians are marked. Abbreviation: HCA, acute histological chorioamnionitis.

There were no differences in the percentiles of birth weights of newborns among women with HCA with fetal (median 52, IQR 33–67) and maternal (median 55, IQR 32–70) inflammatory responses and those with the absence of HCA (median 52, IQR 34–71 *p* = 0.56; Figure 2A), as well as among women with the presence of HCA with (median 52, IQR 33–68) and without (median 55, IQR 32–69) acute inflammation of the amnion and those the absence of HCA (median 52, IQR 34–71 *p* = 0.41; Figure 2B).

No difference in the percentiles of birth weights of newborns was found among women with the presence of HCA with acute inflammation of the amnion, when the women were divided into four subgroups based on the severity of acute inflammation of the amnion [grade I (median 53, IQR 34–73), grade II (median 52, IQR 28–66), grade III (median 48, IQR 30–66), and grade IV (median 50, IQR 41–65); *p* = 0.85; Figure 2C].

No differences were observed in the rates of the birth weights of newborns that were less than or equal to the first, 10th, and 25th percentiles among women with the presence of HCA with fetal and maternal inflammatory responses and women with the absence of HCA (Table 5) and among those with the presence of HCA with and without acute inflammation of the amnion and women with the absence of HCA (Table 5), except the 10th percentile in the subgroups of women with the presence of HCA with fetal and maternal inflammatory responses and women with the absence of HCA (*p* = 0.05)*.* However, the result did not remain significant after adjusting for potential confounders (*p* = 0.08)*.* No differences were observed in the rates of the birth weights of newborns that were less than or equal to the first, 10th, and 25th percentiles among women with the presence of HCA with acute inflammation of the amnion, when the women were divided into four groups based on the severity of acute inflammation of the amnion (Table 5).

**TABLE 5:** The rate of newborns with birth weights, expressed as percentiles derived from the birth weight standards, that were less than or equal to the first, 10th, and 25th percentiles according to: (a) the presence of HCA with fetal and maternal inflammatory responses and the absence of HCA; (b) the presence of HCA with and without acute inflammation of the amnion and the absence of HCA; and (c) severity of acute inflammation of the amnion (grades I–IV).

	≤1 percentile		≤10 percentiles		≤25 percentiles	
a)						
The presence of HCA with fetal inflammatory response (*n* = 343)	0 (0%)	*p* = 0.11	11 (3%)	***p* = 0.05**	55 (16%)	*p* = 0.92
The presence of HCA with maternal inflammatory response (*n* = 151)	0 (0%)		8 (5%)		29 (19%)	
The absence of HCA (*n* = 324)	2 (1%)		21 (6%)		51 (16%)	
b)						
The presence of HCA with acute inflammation of the amnion (*n* = 279)	0 (0%)	*p* = 0.11	12 (4%)	*p* = 0.20	51 (18%)	*p* = 0.41
The presence of HCA without acute inflammation of the amnion (*n* = 215)	0 (0%)		7 (3%)		33 (15%)	
The absence of HCA (*n* = 324)	2 (1%)		21 (6%)		51 (16%)	
c)						
Acute inflammation of the amnion—grade I (*n* = 107)	0 (0%)	-	5 (5%)	*p* = 0.61	20 (19%)	*p* = 0.43
Acute inflammation of the amnion—grade II (*n* = 77)	0 (0%)		4 (5%)		16 (21%)	
Acute inflammation of the amnion—grade III (*n* = 62)	0 (0%)		2 (4%)		12 (19%)	
Acute inflammation of the amnion—grade IV (*n* = 32)	0 (0%)		1 (3%)		3 (10%)	

Variables are presented as number (%) and were compared using Cochran-Armitage test for trend. Statistically significant results are marked in bold.

HCA, acute histological chorioamnionitis.

No result remains significant after the adjustment for potential confounders.

## Discussion

The principal findings of this study are as follows: i) HCA with acute inflammation of the amnion was associated with lower birth weight, expressed as percentiles derived from the estimated fetal weight standards; ii) HCA with acute inflammation of the amnion was related to the highest rate of newborns with a birth weight equal to or below the 25th percentile derived from the estimated fetal weight standards; iii) alteration of fetal growth, expressed as percentiles derived from the estimated fetal weight standards, associated with HCA with acute inflammation of the amnion was dependent on the severity of acute inflammation; and iv) when percentiles were derived from the birth weight standard, no difference in percentiles of the birth weights of newborns was observed among those with the presence of HCA with fetal and maternal inflammatory responses and those with the absence of HCA, as well as among those with the presence of HCA with and without acute inflammation of the amnion and those with the absence of HCA.

Neutrophils are not usually present in the placental tissue and fetal membranes (Kim et al., 2015). Following chemotactic stimulus and its gradient, neutrophils migrate towards the amniotic cavity i) from the intervillous space of the placenta into the chorionic plate and/or ii) from the decidua into fetal membranes (Kim et al., 2015). To develop acute inflammation of the amnion, neutrophils need a strong chemotactic stimulus because they must transmigrate through the entire chorionic layer (Park et al., 2009; Goldstein et al., 2020). This process is time-consuming and requires more than 36 h to develop acute inflammatory changes in the amnion from the first exposure to an inflammatory stimulus (Redline, 2006; Goldstein et al., 2020).

Besides the placentas from pregnancies complicated by PPROM and spontaneous preterm labor, the presence of HCA with acute inflammation of the amnion has also been reported in preterm pregnancies with impaired fetal growth (Salafia et al., 1995). Salafia et al. observed HCA with acute inflammation of the amnion in 37% of the pregnancies with appropriate-for-gestational-age newborns, 38% of the pregnancies with “asymmetric intrauterine growth restriction” and 8% of the pregnancies with “symmetric intrauterine growth restriction” (Salafia et al., 1995).

In this study, newborns from pregnancies with HCA with acute inflammation of the amnion had the lowest birth weight, expressed in percentile derived from the estimated fetal weight standard. This interesting finding was further supported by the fact that the alteration of fetal growth was dependent on the severity of acute inflammation of the amnion. The lowest percentiles and the highest rates of birth weight equal to or less than the 10th and 25th percentiles were found in the women with the most severe form of acute inflammation of the amnion (grade IV). The mechanistic explanation for this observation is unclear. It can be hypothesized that the placenta is affected by various lesions (maternal or fetal vascular malperfusion or chronic villitis), which are responsible for impaired fetal growth, might produce endogenous “danger signals” (e.g., heat shock protein 70, high mobility group box-1, or S100B) (Gazzolo et al., 2006; Zenerino et al., 2017; Lai et al., 2020). These signals lead to the production of chemotactic stimuli such as interleukin (IL)-8, which is followed by the migration of maternal neutrophils into the placenta and/or fetal membranes. When the production of chemotactic stimuli persists long enough, neutrophils can get a sufficient temporal period to reach and infiltrate the amnion. This hypothesis is supported by the following observations: i) placentas from pregnancies with FGR had a higher expression of mRNA for IL-8 (Hahn-Zoric et al., 2002); ii) maternal blood lymphocytes from pregnancies with FGR produce higher levels of IL-8 after stimulation with trophoblast (Raghupathy et al., 2012); iii) maternal serum concentrations of IL-8 are higher in pregnancies with SGA fetuses (Wang et al., 2015); and iv) malarial infection in pregnancy, usually leading to FGR, is associated with a higher expression of IL-8 mRNA in the placenta (Moormann et al., 1999). This hypothesis suggests that placental changes leading to impaired fetal growth are associated with the development of the acute inflammation of the amnion rather than the fetal inflammatory response, as shown in this study. We cannot fully exclude a contribution of fetal membranes on the production of the chemotactic stimuli along with the placenta for the following reasons: i) transporter proteins in the fetal membranes along with nutritional transport system suggests that fetal membranes play an equal role to that of the placenta in drug and nutrients transports (Ganguly et al., 2021; Kammala et al., 2022); ii) endogenous activities in the fetal membranes on cellular level can generate danger signals (Menon and Peltier, 2020; Sheller-Miller and Menon, 2020; Shahin et al., 2021; Shepherd et al., 2021; Tantengco et al., 2021); iii) fetal membranes function can be independent of the placenta and placental involvement (Menon, 2016); iv) fetal growth restriction can increase apoptosis in the chorionic trophoblast cells of fetal membranes and expression of parathyroid-related protein expression in the fetal membranes (Curtis et al., 2000; Murthi et al., 2005); and v) fetal membranes are not the mere extension of the placenta and have their own identity, function and hence, their compromise alone without the placental involvement can be detrimental (Collins et al., 1993; Menon and Moore, 2020). Therefore, functions of fetal membranes might be impaired in pregnancies complicated by the alteration of fetal growth.

The cutoff value of the 10th percentile is widely accepted by obstetricians and pediatricians as a threshold for identifying fetuses/newborns with SGA (DiGiulio et al., 2010; Figueras and Gratacos, 2014). This was the reason why this cutoff, along with the first percentile (to identify newborns with extremely impaired growth) and 25th percentile (to reveal a mild alteration of growth not fulfilling a threshold for SGA), were selected and used in this study. According to the abovementioned results, the highest rate (47%, 89/190) of newborns with having birth weight equal to or less than the 25th percentile was identified in the subset of women with HCA with acute inflammation of the amnion. This observation seems to be clinically relevant because it suggests that underlying placental pathologies associated with impaired fetal growth might be lasting enough to develop acute inflammatory lesions in the amnion but not the restriction of fetal growth that reaches the threshold of the 10th percentile due to subsequent/co-incidental development of PPROM.

Two INTERGROWTH-21st standards (for estimated fetal weight and newborn birth weight) were used in this study to derive percentiles for the birth weights of newborns. Two different growth charts were employed because it is still under debate which standard should be preferred (Marsal et al., 1996; Salomon et al., 2007; Kiserud et al., 2017; Stirnemann et al., 2017; Nicolaides et al., 2018). The main advantage of using the standard for estimated fetal weight is that reference ranges of the estimated fetal weight are representative of the whole population. On the other hand, the reference ranges of birth weight standards, particularly for newborns delivered preterm, suffer from the overrepresentation of pathological pregnancies resulting in iatrogenic or spontaneous preterm delivery in whom impaired placentation might be expected (Nicolaides et al., 2018). This limitation of birth weight standard might be seen mainly in the subset of newborns delivered before 33 weeks of gestation(Villar et al., 2014a). These methodological differences between the charts were observed in this study. For example, the subgroups of women with fetal inflammatory response and acute inflammation of the amnion, with the lowest gestational age at delivery, had lower medians of the percentiles of birth weight derived from the estimated fetal weight standard than when they were derived from the birth weight standard. Taking this into consideration, clinicians should be aware that using a growth chart based on birth weight might not reveal all cases with mildly impaired fetal growth, mainly in lower gestational ages. In light of this fact, it is not surprising that no differences among the assessed subgroups were observed when percentiles derived from the birth weight standard were used. In addition, no differences were observed in the rates of the birth weight equal to or lower than the first, 10th, and 25th percentiles among the subgroups of women with PPROM.

This study has two main strengths. First, the study was conducted on a large homogeneous cohort of Caucasian women with a thoroughly defined phenotype of spontaneous preterm delivery. Second, the assessment of HCA was performed by a single, experienced perinatal pathologist who was blinded to the clinical status of the women. However, there are certain limitations to this study. First, this study covers a 13 years-long interval. During this time, two different management strategies (active management and expectant management) of PPROM pregnancies were used. Therefore, attention was paid to this important confounding factor in this study, and the results were adjusted for the same. Second, no data regarding ultrasonographically estimated fetal weight from the time of delivery or shortly before delivery were available. This shortcoming prevented us from assessing the relationship between the estimated fetal weight, expressed in percentiles derived from the estimated fetal weight standards and the presence of acute inflammatory lesions in the placenta. Third, the histopathological assessment of placental lesions other than HCA (maternal and fetal vascular malperfusion, placental hemorrhage, and chronic villitis) were not systematically performed in the placentas from PPROM pregnancies. Last, the risk factors of the development of fetal and maternal inflammatory responses were not assessed and evaluated in this study.

In conclusion, in this large retrospective cohort study of singleton pregnancies with PPROM, acute inflammation of the amnion was associated with a lower birth weight but only when the percentiles of birth weight were derived from the standards for estimated fetal weight.

## Data Availability

The raw data supporting the conclusion of this article will be made available by the authors, without undue reservation.
